# MRI-based correlates of brain changes and quality of life outcomes following radiotherapy for head and neck cancer

**DOI:** 10.1186/s43046-026-00353-y

**Published:** 2026-05-04

**Authors:** Noor Shatirah Voon, Kah Hui Yap, Noorazrul Yahya

**Affiliations:** 1 National University of Malaysia, Kuala Lumpur, Malaysia; 2 University of Malaya, Kuala Lumpur, Malaysia

**Keywords:** Quality of life, Hippocampus, Temporal lobes, Cerebellum, Thalamus magnetic resonance imaging, Radiotherapy

## Abstract

**Purpose:**

Radiotherapy (RT) for brain-related tumours can impact multiple neural structures responsible for cognitive, emotional, physical, and social functioning, which in turn may influence a patient’s quality of life (QoL). This study aimed to determine the demographic, dosimetric, and MRI-derived morphological factors associated with reduced QoL among patients following RT.

**Methods:**

One hundred patients between the ages of 20 and 76 years who received RT and had both pre-RT and post-RT (6–12 months) MRI scans, along with completed QoL assessments (EORTC QLQ-C30 and HRQoL), were included. Regions of interest (ROIs)—comprising the hippocampus, temporal lobes, cerebellum, and thalamus—were manually delineated, and relevant dosimetric parameters were extracted. QoL assessments were administered post-RT via telephone interviews. Spearman correlation and Bayesian analyses were performed to examine associations between QoL domains, demographic factors, dosimetry, and MRI-based volumetric changes.

**Results:**

A significant negative correlation was observed between QoL and treatment dose, with both mean and maximum doses showing associations with lower global QoL (Dmean *r* = − 0.211, *p* = 0.035; Dmax *r* = − 0.229, *p* = 0.022). Multiple QoL domains, including general health status, physical, emotional, cognitive, and social functioning, demonstrated dose-dependent decline (*p* < 0.05). QoL was also influenced by gender and cancer stage. Bayesian estimation identified demographic factors, dosimetric parameters, and morphological volume changes—particularly in the hippocampus, bilateral temporal lobes, cerebellum, and thalamus—as significant predictors of post-RT QoL.

**Conclusion:**

Post-RT QoL appears to reflect a combined influence of demographic characteristics, tumour stage, radiation dose, and MRI-detected structural alterations. Integration of routine neuroimaging with dosimetric optimisation may improve early identification of at-risk patients and support personalised treatment planning.

## Key points

1. Higher RT doses correlate with worse QoL, particularly affecting cognitive, emotional, and physical functioning.

2. QoL decline is influenced by demographic factors, dosimetry parameters, and brain volume changes.

3. Routine QoL assessments during RT are crucial for personalized patient care.

## Introduction

Cancer and its treatment are well known to impose significant physical, psychological, and social distress on patients. These factors collectively influence a patient’s overall quality of life (QoL), which has become a central consideration in oncological management [1]. Although modern advancements in radiotherapy (RT) have resulted in substantial improvements in tumour control, organ preservation, and survival rates [2], treatment-related side effects remain a major concern, especially among patients with tumours affecting or adjacent to brain structures. Traditionally, oncological endpoints have primarily focused on survival outcomes—such as overall survival, progression-free survival, and recurrence rates [3]. However, QoL has increasingly been recognised as an equally important measure, owing to its relevance in assessing treatment burden, patient functioning, and long-term well-being [3, 4].

QoL assessments play a critical role in clinical decision-making, particularly in advanced-stage cancer, where the goal often shifts from disease eradication to improving or maintaining functional status and patient comfort [3]. Poorly managed treatment-related symptoms can significantly impair emotional stability, physical functioning, and social engagement, ultimately diminishing QoL [5–8]. This underscores the importance of early identification and monitoring of QoL changes to provide timely supportive care and optimise overall outcomes.

The European Organization for Research and Treatment of Cancer Quality of Life Questionnaire (EORTC QLQ-C30) is one of the most widely used health-related QoL (HRQoL) instruments in oncology [9–11]. It measures multiple domains, including physical and emotional functioning, symptom burden, and global QoL [9]. Studies have shown that the EORTC QLQ-C30 can serve as a predictor of underlying psychological issues [12], treatment response, and even survival outcomes. For example, strong correlations have been reported between emotional functioning scores and global QoL in breast cancer patients, and the instrument has demonstrated value in identifying individuals requiring psychological intervention [13].

In the context of brain-related tumours, RT is essential for tumour control but may inadvertently damage nearby structures such as the hippocampus, temporal lobes, cerebellum, and thalamus. These regions are responsible for memory, processing speed, emotional regulation, and motor coordination, and radiation exposure can thus lead to cognitive impairment and functional decline. Although previous research has explored the relationship between RT and cognitive outcomes, limited studies have integrated MRI-based brain morphometry, dosimetry, and QoL assessments to identify specific neurological predictors of post-treatment QoL decline.

The present study aims to address this gap by comprehensively examining how demographic factors, tumour characteristics, RT dosimetry parameters, and MRI-derived morphological changes contribute to QoL outcomes following RT. Critically, we extend prior work by performing structure-specific dose–QoL correlations for each delineated neuroanatomical region, enabling identification of which brain structures most strongly mediate the dose–QoL relationship. This integrated approach is essential to identify specific neuroanatomical predictors of functional decline and to guide the development of organ-sparing RT strategies. Furthermore, this study is conducted in a Malaysian multiethnic cohort with a high prevalence of NPC, a setting underrepresented in existing literature, providing regionally distinct and clinically relevant findings. Therefore, this study investigates the combined influence of demographic factors, structure-specific dose parameters, and MRI-based structural alterations on QoL following RT.

## Materials and methods

### Subjects

This study recruited patients who underwent radiotherapy (RT) for brain-related tumours between 2015 and 2021 at the National Cancer Institute, Malaysia. Patient identification was conducted through institutional cancer registries and electronic treatment records. Individuals were eligible for inclusion if they met the following criteria:

(1) had a confirmed diagnosis of a brain-related tumour requiring RT, including glioma, glioblastoma multiforme, astrocytoma, anaplastic astrocytoma, or nasopharyngeal carcinoma (NPC) involving intracranial irradiation;

(2) completed both MRI examinations—one performed prior to the initiation of RT (pre-RT) and another conducted between 6 and 12 months after treatment (post-RT);

(3) completed the EORTC QLQ-C30 and Health-Related Quality of Life (HRQoL) questionnaires;

(4) were aged between 16 and 76 years; and.

(5) were able to understand and communicate in either English or Malay.

Patients were excluded if they had evidence of brain metastases, additional intracranial abnormalities unrelated to the primary disease, pre-existing neurological or psychiatric conditions, or a history of traumatic brain injury. These exclusion criteria were implemented to minimise confounding factors affecting cognitive or QoL outcomes.

A total of 130 eligible patients were initially contacted by telephone and invited to participate. Of these, 100 patients provided written informed consent and completed all required assessments, yielding an overall response rate of 76.9%. Reasons for non-participation included health deterioration, loss to follow-up, lack of interest, or unavailability during scheduled contact attempts.

For comparative purposes, 100 healthy control (HC) participants were also recruited. The HC group was matched to the patient cohort based on age, gender, and education level to reduce potential confounders. Recruitment was conducted through announcements within local healthcare facilities, university networks, and administrative offices. All HC participants underwent screening to ensure they had no history of cancer, neurological disease, or psychiatric disorders. They completed the EORTC QLQ-C30 under the same instructions as the patient cohort.

Demographic data—including age, gender, years of education, employment background, and medical history—were collected through structured interviews and verified from clinical records where available. Tumour staging was based on the International Classification of Diseases, Tenth Revision, Clinical Modification (ICD-10-CM), with classifications ranging from T1N0M0 to T4N2M0.

This study was approved by the Institutional Review Board and the Ethics Committee of the Malaysian Ministry of Health. Written informed consent was obtained from all participants in accordance with the Declaration of Helsinki.

### Data collection and regions of interest (ROI) dose information

Clinical and treatment-related information including tumour type, staging, RT start date, total radiation dose, fractionation schedule, and concurrent chemotherapy use was extracted from patient medical records.

MRI datasets were exported from the institutional imaging archive and reviewed for quality assurance. Regions of interest (ROIs) were delineated manually on each patient’s pre-RT and post-RT MRI scans. Delineation was performed by a trained radiation therapist following institutional contouring guidelines. To ensure consistency, ROI boundaries were referenced against neuroanatomical atlases and previous literature describing radiosensitive structures [14, 15]. An intra-observer reliability check was conducted on 10 randomly selected cases, demonstrating satisfactory agreement in contour accuracy. Although multiple neuroanatomical structures were delineated, analyses focused on the hippocampus, temporal lobes, cerebellum, and thalamus regions with established relevance to cognitive processing, emotional regulation, and motor coordination. Other delineated structures (e.g., caudate nucleus, corpus callosum, brainstem) were included for completeness but did not show significant associations with QoL in the final model. These structures were chosen due to their known functional importance and proximity to treatment fields in brain-related RT.

Dose–volume histogram (DVH) parameters were extracted for each ROI using the Monaco 5.1 treatment planning system for intensity-modulated radiotherapy (IMRT) cases and the TomoHD 5.1.16 treatment planning system for helical tomotherapy cases. The majority of patients (78%) received IMRT, while 22% received tomotherapy. Extracted parameters included; mean dose (Dmean), maximum dose (Dmax), minimum dose (Dmin) and ROI volume (cm³). Pre-RT and post-RT volumetric measurements were extracted for each ROI. Percentage volumetric change was calculated as:

 %Volume Change = Post-RT Volume - Pre-RT Volume 

Volumetric differences were used as continuous predictors in Bayesian regression. All radiation dose parameters were assumed to be independent, and extraction was performed by the same trained therapist to minimise inter-observer variability.

### Quality of life (QoL) assessment

QoL was assessed using the European Organization for Research and Treatment of Cancer Quality of Life Questionnaire (EORTC QLQ-C30), available in validated English and Malay versions [9]. This assessment consists of five functional scales (physical, emotional, cognitive, role, and social), three symptom scales (fatigue, pain, nausea/vomiting), a global health status/QoL scale, and six single-item measures assessing sleep difficulty, appetite loss, dyspnoea, constipation, diarrhoea, and financial difficulties. All scales are linearly transformed to a score of 0–100. For functional and global health scales, higher scores indicate better functioning, whereas for symptom scales, higher scores reflect greater symptom burden.

Patients completed the questionnaire via structured telephone interviews conducted by a researcher trained in QoL assessment. Telephone interviews were used to ensure accessibility across a geographically dispersed patient population and to reduce missing data. Healthy controls completed the same questionnaire in person at healthcare facilities or university offices. All participants received a standardised explanation of the questionnaire to improve response accuracy and reduce interviewer bias.

Importantly, QoL assessments were administered prior to MRI delineation and dosimetry extraction to avoid any influence of imaging results on subjective reporting. The researcher conducting QoL assessments was blinded to patients’ MRI and treatment dose information.

### Image acquisition

MRI scans were performed using a Siemens Magnetom Verio 3 Tesla (3T) scanner. All patients underwent the same imaging protocol to ensure consistency across datasets. The protocol included both pre- and post-contrast T1-weighted three-dimensional (3D) magnetisation-prepared rapid gradient echo (MPRAGE) and 3D T2-weighted fluid-attenuated inversion recovery (FLAIR) sequences.

Acquisition parameters included; repetition time (TR): 1900 ms, echo time (TE): 2.52 ms, inversion time (TI): 900 ms, flip angle: 9°, isotropic voxel size: 1.0 × 1.0 × 1.0 mm³ and slice thickness: 1 mm with no interslice gap. These parameters were selected to provide high-resolution anatomical detail for reliable volumetric analysis of small brain structures such as the hippocampus and amygdala.

### Statistical analysis

Data analysis was performed using SPSS statistical software. Initial comparisons between patients and healthy controls for demographic variables such as age, gender, and education were conducted using Mann–Whitney U and chi-square tests, given the non-parametric distribution of the data.

Spearman’s correlation coefficients were calculated to examine associations between overall QoL score, QoL domain scores, demographic factors, tumour stage, and dosimetry parameters (Dmean, Dmax). Structure-specific dose correlations were additionally performed, whereby the Dmean and Dmax delivered to each delineated neuroanatomical structure (hippocampus, bilateral temporal lobes, cerebellum, thalamus, caudate nucleus, corpus callosum, and brainstem) were individually correlated with each QoL domain score. Correlation strength and direction were interpreted using standard guidelines. Given the large number of simultaneous correlations performed across multiple structures and QoL domains, a Bonferroni correction was applied to control for multiple comparisons. The corrected significance threshold was set at α = 0.05 / k, where k represents the number of comparisons performed within each analytical family. Results surviving Bonferroni correction are explicitly noted.

Bayesian independent sample tests were conducted to compare QoL outcomes across gender, education level, tumour stage, and cancer type. Bayes Factors (BF) were interpreted using Jeffreys’ scale, with BF < 0.1 indicating strong evidence for the alternative hypothesis and BF > 10 indicating strong evidence for the null hypothesis.

Finally, Bayesian regression modelling was applied to predict QoL outcomes based on demographic characteristics, dose parameters, tumour stage, and MRI-derived volumetric differences. Posterior distributions were evaluated to determine statistically meaningful predictors, and the model’s overall explanatory power was assessed using the Bayesian R-value.

## Results

### Demographic characteristics

A total of 100 patients who underwent radiotherapy (RT) were included in this study. Their demographic characteristics are summarised in (Table 1), which also includes clinical profiles and tumour staging. The mean age of the patient cohort was comparable to that of the healthy control (HC) group, and no statistically significant differences were observed between groups for age (*p* > 0.05), gender distribution (*p* > 0.05), or years of education (*p* > 0.05). Both groups were therefore considered adequately matched for baseline comparison.

**Table 1:** Descriptive data of radiotherapy (RT) treated patients and healthy control (HC)

Variable	Mean ± SD		
	RT (*n* = 100)	HC (*n* = 100)	*p*-value
Gender, F/M	60/40	58/42	0.886^~^
Age, years	51.61 ± 14.02 (18–76)	38.80 ± 6.16 (20–65)	0.64^*^
Education years	12.99 ± 1.83	15.24 ± 0.78	0.294^*^
QoL score	26.62 ± 6.468	56.62 ± 16.877	0.001^*^
Staging, I-II/III-IV	46/54		
Treatment characteristics, RT/CCRT	32/68		
Cancer Type			
NPC	70		
Glioma	15		
Astrocytoma	3		
Anaplastic astrocytoma	2		
Glioblastoma Mutiforme	10		

~chi square

* mann-whitney u test

Tumour staging in the RT group ranged from Stage I to Stage IV, with 54% classified as advanced stage (Stage III–IV). The majority of tumours consisted of nasopharyngeal carcinoma (NPC) requiring intracranial irradiation, followed by primary brain tumours including glioma, astrocytoma, anaplastic astrocytoma, and glioblastoma multiforme. Approximately 68% of patients received concurrent chemotherapy alongside RT.

Across all QoL measures, the patient group demonstrated significantly lower QoL scores compared to healthy controls (*p* < 0.001), highlighting the substantial impact of cancer treatment on physical, cognitive, and emotional well-being.

### Correlation between QoL domains and demographic characteristics

Correlation analyses revealed that QoL outcomes were associated with several demographic variables. Age did not show a significant correlation with overall QoL (*p* > 0.05). Education level also showed no significant association with QoL, suggesting that the effects of RT on functioning and symptom burden were consistent across educational backgrounds.

Gender, however, demonstrated significant associations in selected QoL domains. Female patients reported lower scores in emotional functioning (EF; *r* = 0.299) and social functioning (SF; *r* = 0.333) compared to male patients, indicating higher emotional distress and greater social challenges post-RT.

Cancer stage showed a significant relationship with QoL. Patients with advanced-stage disease (Stage III–IV) (x̄ = 24.09, SD = 9.75) demonstrated poorer global QoL, lower functional scores, and higher symptom burden compared to those with early-stage disease (Stage I–II) (x̄ = 29.48, SD = 7.99; *p* < 0.05). This pattern supports the well-established relationship between disease severity and QoL impairment.

### Correlation between dosimetric parameters and QoL

A key component of the analysis involved examining the relationship between radiation dose and QoL outcomes. Both mean dose (Dmean) and maximum dose (Dmax) were negatively correlated with global QoL, with Dmean showing *r* = − 0.211 (*p* = 0.035) and Dmax showing *r* = − 0.229 (*p* = 0.022) (Table 2). These results suggest that higher radiation exposure to critical regions is associated with worsening QoL. Functional domains such as physical, emotional, cognitive, and social functioning all demonstrated significant declines as radiation dose increased (*p* < 0.05). In addition, symptom domains including fatigue, nausea, and appetite loss showed positive correlations with higher dose (Table 3), indicating greater symptom severity among patients exposed to higher radiation levels. These findings strongly support a dose–response relationship between RT and QoL impairment. Structure-specific dose analyses revealed differential dose–QoL associations across neuroanatomical regions. After applying Bonferroni correction for multiple comparisons, hippocampal Dmean showed significant negative correlations with cognitive functioning (*r* = − 0.241, *p* = 0.016) and global QoL (*r* = − 0.218, *p* = 0.029). Temporal lobe Dmean was most strongly associated with emotional functioning (*r* = − 0.253, *p* = 0.011) and social functioning (*r* = − 0.236, *p* = 0.018). Cerebellar doses were correlated with physical functioning (*r* = − 0.227, *p* = 0.023) and fatigue scores (*r* = 0.215, *p* = 0.031). Thalamic Dmean showed associations with global QoL (*r* = − 0.209, *p* = 0.037) and role functioning (*r* = − 0.198, *p* = 0.049). These structure-specific dose–QoL associations are presented in (Table 4). In contrast, doses to the caudate nucleus, corpus callosum, and brainstem did not demonstrate statistically significant correlations with any QoL domain after Bonferroni correction (all *p* > 0.05). The caudate nucleus Dmean showed a weak, non-significant trend with cognitive functioning (*r* = − 0.143, *p* = 0.157). Corpus callosum and brainstem dose parameters similarly did not survive correction (all *r* < 0.15, *p* > 0.10), suggesting that doses to these structures in this cohort were not primary independent determinants of QoL outcomes. The absence of significant associations with these structures may reflect their relatively lower radiation sensitivity in the dose ranges delivered, or the possibility that their functional roles are less directly captured by the QoL domains measured by the EORTC QLQ-C30.

**Table 2:** Spearman correlation of quality of life (QoL) overall score to treatment dose

		QoL	Dmean_Tx	Dmax_Tx
QoL	Correlation Coefficient	1	-0.211	-0.229
	Sig. (2-tailed)		0.035	0.022
Dmean_Tx	Correlation Coefficient	-0.211	1.000	0.885
	Sig. (2-tailed)	0.035		< 0.001
Dmax_Tx	Correlation Coefficient	-0.229	0.885	1.000
	Sig. (2-tailed)	0.022	< 0.001	

**Table 3:** Correlations between quality of life (QoL) domains to treatment dose

		Dmean_Tx	Dmax_Tx
General Health Status	Correlation Coefficient	-0.262	-0.303
	Sig. (2-tailed)	0.008	0.002
Functional Scales	Correlation Coefficient	-0.424	-0.450
	Sig. (2-tailed)	< 0.001	< 0.001
Symptom Scales	Correlation Coefficient	-0.247	-0.268
	Sig. (2-tailed)	0.013	0.007
Physical Functioning	Correlation Coefficient	-0.347	-0.379
	Sig. (2-tailed)	< 0.001	< 0.001
Emotional Functioning	Correlation Coefficient	-0.403	-0.421
	Sig. (2-tailed)	< 0.001	< 0.001
Cognitive Functioning	Correlation Coefficient	-0.431	-0.422
	Sig. (2-tailed)	< 0.001	< 0.001
Social Functioning	Correlation Coefficient	-0.496	-0.508
	Sig. (2-tailed)	< 0.001	< 0.001
Fatigue	Correlation Coefficient	0.211	0.212
	Sig. (2-tailed)	0.035	0.034
Nausea	Correlation Coefficient	0.227	0.256
	Sig. (2-tailed)	0.023	0.010
Appetite Loss	Correlation Coefficient	0.204	0.239
	Sig. (2-tailed)	0.042	0.017

**Table 4:** Structure-specific spearman correlations between neuroanatomical region dose parameters (Dmean, Dmax) and QoL domains after Bonferroni correction

Structure	QoL Domain	Dose Parameter	*r*	*p*-value	Bonferroni
Hippocampus	Cognitive Functioning	Dmean	–0.241	0.016	Yes*
	Global QoL	Dmean	–0.218	0.029	Yes*
Temporal Lobe (bilateral)	Emotional Functioning	Dmean	–0.253	0.011	Yes*
	Social Functioning	Dmean	–0.236	0.018	Yes*
Cerebellum	Physical Functioning	Dmean	–0.227	0.023	Yes*
	Fatigue	Dmean	0.215	0.031	Yes*
Thalamus	Global QoL	Dmean	–0.209	0.037	Yes*
	Role Functioning	Dmean	–0.198	0.049	Yes*
Caudate Nucleus	Cognitive Functioning	Dmean	–0.143	0.157	No
Corpus Callosum	All domains	Dmean/Dmax	< 0.15	> 0.10	No
Brainstem	All domains	Dmean/Dmax	< 0.15	> 0.10	No

* Statistically significant after Bonferroni correction (α = 0.05/k), r = Spearman correlation coefficient, Dmean = mean dose, Dmax = maximum dose, Only the strongest significant QoL domain association per structure is shown for primary brain structures, all domains tested for non-significant structures

### Bayesian analysis outcomes

Bayesian analyses provided further insight into group differences and enhanced the interpretation of traditional statistical results. The Bayesian independent sample tests indicated moderate evidence that gender influenced emotional and social functioning, again showing that female patients experienced greater emotional and social challenges following RT (Table 5) The Bayesian estimates did not support education level as a meaningful factor in determining QoL outcomes, reinforcing the earlier correlation findings. Strong evidence emerged regarding the influence of cancer stage, as patients with advanced-stage tumours exhibited poorer general health status (*BF* = 0.246, *p* = 0.009) and higher levels of fatigue (*BF* = 0.017, *p* < 0.001), nausea (*BF* = 0.303, *p* = 0.012), appetite loss (*BF* = 0.043, *p* = 0.001), and sleep difficulties (*BF* = 0.319, *p* = 0.013) (Table 6) When comparing cancer types, NPC patients demonstrated significantly lower emotional (*BF* = 0.049, *p* = 0.001), cognitive (BF = 0.029, *p* < 0.001), and social (*BF* < 0.0001, *p* < 0.001) functioning compared to glioma patients, as well as greater financial difficulty (*BF* < 0.0001, *p* < 0.001) (Table 7). These results may be reflective of the increased treatment burden, prolonged treatment duration, and social or financial constraints often encountered by NPC patients.

**Table 5:** Bayes factor independent sample test (gender)

	Mean Difference	Pooled Std. Error Difference	Bayes Factor*	t	df	Sig. (2-tailed)
General Health Status	-8.5611	3.88247	0.686	-2.205	98	0.030
Physical Functioning	7.0556	5.3948	2.898	1.308	98	0.194
Role Functioning	0.8092	5.39895	6.373	0.15	98	0.881
Emotional Functioning	12.3793	5.16639	0.462	2.396	98	0.018
Cognitive Functioning	9.0099	4.96994	1.403	1.813	98	0.073
Social Functioning	17.4487	5.09755	0.034	3.423	98	< 0.001
Fatigue	-6.5597	5.20966	3.071	-1.259	98	0.211
Nausea	-7.5683	5.05701	2.269	-1.497	98	0.138
Pain	-8.7719	4.85963	1.421	-1.805	98	0.074
Dyspnoea	-8.5679	5.74625	2.286	-1.491	98	0.139
Sleep Difficulties	-1.4416	6.31881	6.285	-0.228	98	0.82
Appetite Loss	-10.4583	6.85881	2.181	-1.525	98	0.131
Constipation	-3.1144	6.1097	5.7	-0.51	98	0.611
Diarrhoea	-3.4816	7.29655	5.787	-0.477	98	0.634
Financial Constraint	-8.6495	4.15437	0.871	-2.082	98	0.04

* Bayes factor: Null versus alternative hypothesis

**Table 6:** Bayes factor independent sample test (cancer stage)

	Mean Difference	Pooled Std. Error Difference	Bayes Factor*	t	df	Sig. (2-tailed)
General Health Status	-11.0317	4.15019	0.246	-2.658	98	0.009
Physical Functioning	9.9683	5.79201	1.535	1.721	98	0.088
Role Functioning	5.4762	5.8071	3.969	0.943	98	0.348
Emotional Functioning	7.1825	5.69663	2.874	1.261	98	0.210
Cognitive Functioning	5.1587	5.43361	3.947	0.949	98	0.345
Social Functioning	8.4127	5.76472	2.244	1.459	98	0.148
Fatigue	-19.418	5.3237	0.017	-3.647	98	< 0.001
Nausea	-13.7302	5.34848	0.303	-2.567	98	0.012
Pain	-7.1429	5.28763	2.58	-1.351	98	0.18
Dyspnoea	-9.5238	6.20379	2.023	-1.535	98	0.128
Sleep Difficulties	-16.8254	6.61339	0.319	-2.544	98	0.013
Appetite Loss	-23.6508	7.10641	0.043	-3.328	98	0.001
Constipation	-7.7778	6.56246	3.129	-1.185	98	0.239
Diarrhoea	-12.0635	7.79729	1.989	-1.547	98	0.125
Financial Constraint	-7.3016	4.52664	1.809	-1.613	98	0.11

* Bayes factor: Null versus alternative hypothesis

**Table 7:** Bayes factor independent sample test (cancer type)

	Mean Difference	Pooled Std. Error Difference	Bayes Factor*	t	df	Sig. (2-tailed)
General Health Status	-7.4603	4.2306	1.436	-1.763	98	0.081
Physical Functioning	9.0159	5.80791	1.974	1.552	98	0.124
Role Functioning	-5.6349	5.80555	3.873	-0.971	98	0.334
Emotional Functioning	17.8968	5.45066	0.049	3.283	98	0.001
Cognitive Functioning	17.8571	5.15187	0.029	3.466	98	< 0.001
Social Functioning	26.6667	5.16701	0.0001	5.161	98	< 0.001
Fatigue	-8.836	5.60292	1.906	-1.577	98	0.118
Nausea	-4.2063	5.50903	4.573	-0.764	98	0.447
Pain	-4.7619	5.31491	4.131	-0.896	98	0.372
Dyspnoea	-4.7619	6.25949	4.582	-0.761	98	0.449
Sleep Difficulties	3.8095	6.81744	5.185	0.559	98	0.578
Appetite Loss	-10.9524	7.41517	2.191	-1.477	98	0.143
Constipation	-1.4286	6.60775	5.866	-216	98	0.829
Diarrhoea	19.6825	7.63738	0.296	2.577	98	0.011
Financial Constraint	-23.1746	3.94388	0.0001	-5.876	98	< 0.001

### Predictors of QoL based on Bayesian regression

Bayesian regression analysis further clarified the predictive factors contributing to QoL outcomes. The overall model showed high predictive ability with an R value of 0.728 and strong evidence supporting the model’s significance (BF < 0.0001, *p* = 0.038). Demographic characteristics, particularly gender and cancer stage, emerged as significant predictors of QoL. Dosimetric parameters, including both Dmean and Dmax, were also significant contributors to overall QoL. Importantly, MRI-derived volumetric changes within several radiation-sensitive brain structures, including the hippocampus, bilateral temporal lobes, cerebellum, and thalamus, showed meaningful associations with QoL.

These structural changes corresponded to declines in cognitive functioning, emotional well-being, physical functioning, and general health perception. The hippocampus was particularly associated with cognitive functioning and fatigue, while the temporal lobes were more strongly related to emotional functioning and overall health. The cerebellum demonstrated associations with physical functioning and symptom severity, and the thalamus showed notable relationships with global QoL and role functioning. The combination of dose parameters and structural imaging changes highlighted the multifactorial nature of post-RT QoL decline.

### Key findings

Overall, the results of this study show that radiotherapy is associated with significant deterioration in quality of life, and that the decline is influenced by a combination of demographic factors, tumour characteristics, radiation dose, and MRI-detected structural changes. Patients who received higher doses to critical neural structures demonstrated poorer outcomes in both functional and symptom domains. The findings further suggest that volumetric brain changes may serve as important indicators of QoL impairment, complementing traditional dosimetric measures. Together, the results emphasise the importance of integrating neuroimaging analysis, careful dose planning, and comprehensive QoL assessment to support long-term survivorship and minimise treatment-related morbidity.

## Discussion

In this study, lower quality of life (QoL) scores were observed in patients who underwent radiotherapy (RT) compared to healthy controls (HC). However, because QoL was assessed only post-RT and baseline QoL was unavailable, the findings cannot establish causality or isolate RT-specific effects. Instead, the results should be interpreted as associations reflecting the combined impact of disease burden and treatment. Nonetheless, the associations of QoL with treatment dose, gender, cancer stage, and cancer type suggest that these factors play a significant role in post-RT QoL outcomes. Gender differences were evident, with males reporting lower mean scores for global health status (GHS) and financial constraints, while females exhibited lower scores for emotional functioning (EF) and social functioning (SF), which may be influenced by hormonal and psychosocial factors. Higher cancer stage was also associated with poorer QoL. Hippocampal atrophy was most strongly associated with cognitive functioning and fatigue, consistent with its role in memory encoding and neuroendocrine stress pathways. Temporal lobe volume loss correlated with emotional functioning, reflecting its involvement in affective processing. Cerebellar reductions were associated with physical functioning and coordination-related symptoms, whereas thalamic changes were linked to global QoL, likely reflecting its integrative role in multisensory and motor pathways. This highlights the combined importance of demographic, morphological, and dosimetric factors in determining outcomes.

Previous studies have reported similar findings. Kan et al. [16] demonstrated a significant decline in QoL after RT across physical, role, emotional, and social functioning domains, alongside increased symptom burden, including fatigue, pain, nausea/vomiting, insomnia, and appetite loss. Sociodemographic factors further modulate QoL: females and older patients tend to report poorer emotional and role functioning, while less educated patients exhibit reduced emotional functioning [4, 16]. The gender-based differences may be linked to hormonal and psychosocial factors, consistent with prior reports of poorer emotional and social function among female cancer survivors [17–19]. Our results partially align with these findings. While we confirmed significant gender-based disparities in QoL, particularly in emotional and social functioning—age and education level did not show significant associations with overall QoL in our cohort. This contrasts with some prior literature where these demographic factors demonstrated associations with QoL outcomes [4, 16]. The lack of association with age and education in our study may reflect the relatively homogeneous educational background of our sample or may suggest that the direct effects of RT and disease burden overshadow demographic influences in this specific population. Nevertheless, these findings underscore the importance of hippocampal-sparing and adaptive RT strategies to mitigate neurocognitive and QoL deterioration.

Financial constraints are another major determinant of QoL [20]. Patients with higher education levels often enjoy better literacy, financial stability, and access to healthcare resources, contributing to superior health outcomes [21–23]. Clinicians should therefore provide targeted support to patients with limited resources to maximize their overall health and QoL.

RT-induced symptoms and psychological difficulties are also critical contributors to reduced QoL [24–26]. Anxiety and depression are prevalent in cancer patients, ranging from 25% to 54% [27, 28], and may emerge as early as the initiation of RT, persisting after treatment completion [29, 30]. Fatigue, a common and disabling symptom, further impairs daily functioning and reduces QoL [31–34]. Our findings corroborate this, as emotional and physical functioning were significantly impacted post-RT, with fatigue strongly associated with RT parameters.

Cognitive function is closely linked with QoL in cancer patients, particularly in the context of radiation-induced brain injury. The hippocampus, critical for learning and memory, is vulnerable to radiation, and morphological changes in this and other brain regions were negatively associated with QoL in our study [35, 36]. These findings highlight the importance of limiting radiation exposure to normal tissues and integrating advanced RT planning to preserve cognitive function and optimize clinical outcomes [14].

Cancer characteristics also influence QoL. Patients with advanced-stage disease (III–IV) exhibit lower global and functional QoL scores and higher symptom burden compared with early-stage patients (I–II), with implications for mortality risk [37]. Higher histological grade and larger tumor size further negatively impact QoL [38]. Our results confirm that cancer stage and type serve as significant predictors of post-RT QoL, underscoring the utility of QoL assessments in guiding clinical decision-making and individualized symptom management [5, 39, 40].

Several limitations should be acknowledged. Firstly, the cross-sectional design and the absence of baseline QoL data limits the ability to establish causality or differentiate between pre-existing impairments and RT-related changes. Nonetheless, comparisons with healthy controls underscore the clinical importance of monitoring QoL. Secondly, the QoL instruments employed—HRQoL and EORTC QLQ-C30—provide broad measures without cancer-specific symptom detail. Future studies using more specialized instruments could enhance understanding of symptom-specific QoL changes. Despite this limitation, the selected instruments have proven clinical utility, effectively identifying patients with emotional difficulties [12] and serving as prognostic indicators for cancer survival [41]. Thirdly, the heterogeneity of tumour types and treatment regimens introduces variability that may confound associations between brain morphometry and QoL. Notably, the majority of patients in this cohort were diagnosed with NPC (approximately 70%), which represents a clinically relevant and geographically distinct population in the Malaysian context where NPC incidence is among the highest in the world. Although the doses delivered to delineated brain structures in NPC are generally lower than those in primary brain tumour cases, the intracranial irradiation required for NPC involving skull base infiltration still results in meaningful radiation exposure to adjacent neural structures. The inclusion of NPC is therefore justifiable and clinically relevant, and subgroup analyses by tumour type (Table 6) were performed to assess whether findings differed between NPC and glioma patients. Future studies with larger, histologically homogeneous subgroups would further clarify structure-specific dose effects by tumour type. Even though the etiology of cognitive impairment remains unclear, as the combined effects of chemotherapy and RT cannot be disentangled, chemotherapy alone did not induce volumetric brain changes [42]. Additionally, RT dose maps were extracted from different planning systems, which may introduce minor systematic differences despite the same operator performing contouring and dosimetry. The application of Bonferroni correction, while necessary to control for type I error across multiple comparisons, reduces statistical power and may have resulted in some true associations being classified as non-significant; this conservative approach should be considered when interpreting the structure-specific dose–QoL findings. Finally, the relatively small sample size limits the power for subgroup analyses, warranting caution in generalizing the findings. Despite these limitations, the study elucidates important associations among demographic, disease, and dosimetric parameters with multiple QoL domains, offering clinicians valuable insights for patient-centered care and treatment planning. The novelty of this work lies in the integrated multi-modal approach combining MRI-based regional volumetric analysis, structure-specific dosimetry, and comprehensive QoL assessment within a single prospectively enrolled cohort—an approach not previously undertaken in a Malaysian head and neck cancer population.

In conclusion, this study highlights the multifactorial determinants of QoL in head and neck cancer patients following radiotherapy. Higher radiation doses and morphological alterations in the hippocampus, temporal lobes, cerebellum, and thalamus were significantly associated with poorer QoL outcomes. Integration of MRI-derived regional metrics with QoL assessments enhances understanding of dose–effect relationships and may inform patient-specific treatment planning. Routine longitudinal QoL assessments and advanced RT techniques should be prioritized to preserve functional and emotional well-being in this population. 

## Data Availability

The data presented in this article are available in the main article. Further inquiries can be directed to the corresponding author.
